# SealedGRID: Secure and Interoperable Platform for Smart GRID Applications †

**DOI:** 10.3390/s21165448

**Published:** 2021-08-12

**Authors:** George Suciu, Mari-Anais Sachian, Alexandru Vulpe, Marius Vochin, Aristeidis Farao, Nikolaos Koutroumpouchos, Christos Xenakis

**Affiliations:** 1 R & D Department, Beia Consult International, 041386 Bucharest, Romania; george@beia.ro (G.S.); anais.sachian@beia.ro (M.-A.S.); 2 Telecommunications Department, University Politehnica of Bucharest, 61071 Bucharest, Romania; alex.vulpe@radio.pub.ro; 3 Department of Digital Systems, University of Piraeus, 18534 Piraeus, Greece; arisfarao@unipi.gr (A.F.); nikoskoutr@ssl-unipi.gr (N.K.); xenakis@unipi.gr (C.X.)

**Keywords:** smart grid, attribute-based access control, eXtensible Access Control Markup Language, Abbreviated Language for Authorization

## Abstract

Recent advancements in information and communication technologies (ICT) have improved the power grid, leading to what is known as the smart grid, which, as part of a critical economic and social infrastructure, is vulnerable to security threats from the use of ICT and new emerging vulnerabilities and privacy issues. Access control is a fundamental element of a security infrastructure, and security is based on the principles of less privilege, zero-trust, and segregation of duties. This work addresses how access control can be applied without disrupting the power grid’s functioning while also properly maintaining the security, scalability, and interoperability of the smart grid. The authentication in the platform presumes digital certificates using a web of trust. This paper presents the findings of the SealedGRID project, and the steps taken for implementing Attribute-based access control policies specifically customized to the smart grid. The outcome is to develop a novel, hierarchical architecture composed of different licensing entities that manages access to resources within the network infrastructure. They are based on well-drawn policy rules and the security side of these resources is placed through a context awareness module. Together with this technology, the IoT is used with Big Data (facilitating easy handling of large databases). Another goal of this paper is to present implementation and evaluations details of a secure and scalable security platform for the smart grid.

## 1. Introduction

Electricity generation accounts for the second-highest amount of greenhouse gas emissions and nearly 63 percent of electricity is generated by fossil fuel combustion, primarily coal and natural gas [1]. Greenhouse gases absorb heat and boost global warming and almost all greenhouse gas increase in the atmosphere over the last 150 years has been attributed to human activity. The evolution of information and communication technologies (ICT) has found its way into the electrical grid and the smart grid represents an opportunity to use new communication systems and information to reshape the traditional electrical power system. However, any significant change provided to the conventional power system involves careful justification and is costly given the size of investment made in it over the years and the vast size of the power system. Thus, the smart grid [2] has been devised as representing an intelligent, responsive, and efficient electrical system, using ICT to bring central monitoring, control, and optimization of the power grid. Smart grids inherit the security threats of the ICT world when applied to economic infrastructure and real-life [3]. Lately, researchers have highlighted the challenge of preserving the smart grid from security threats, which can have a considerable impact on both the power system and human life. The smart grid infrastructure needs to be defended against threats such as privacy issues, the increasing number of intelligent devices, power systems lifetime, and implicit trust between traditional power devices, to name a few. A potential attack against a smart grid system [4] can lead to failures, ranging from the destruction of other interconnected critical infrastructures (e.g., gas, water, and transportation) to loss of human lives [5]. Moreover, the migration of ICT security solutions to the smart grid is done over a long period of time. In this situation, new approaches are needed. Within SealedGRID, security is achieved by combining trusted applications with authorization and authentication components.

Trusted Execution Environment (TEE) [6] represents a sandbox which executes applications in a secure context named trusted applications. Normal applications are usually isolated from the TEE providing a safe environment and thus malicious software won’t harm any sensitive data stored on the device stored in the TEE or used by those of applications. ARM TrustZone provides an implementation of TEE, whereas an ARM processor is often seen in mobile phones.

One of the main research challenges for this work are the integration of authorization and authentication components together with TEE. Further, their integration will be used in the experiments based on following privacy requirements in order to make secure the connection and sensitive data in a smart grid System [7], such as:

- Identity privacy
- Location privacy
- Unlinkability
- Minimum data disclosure
- Privacy—preserving data aggregation.

This paper is organized as follows: Section 2 briefly describes the related work regarding the SealedGRID platform and its envisioned achievements, introducing the challenges and opportunities in smart grids. Section 3 describes the proposed SealedGRID architecture, introducing the main components and methods, and Section 4 presents the authorization component. Section 5 presents the methodology for a trusted computing environment, Section 6 presents the results in evaluating the reference implementation of the SealedGRID authorization and policy enforcement framework and the TEE component, while Section 7 draws conclusions.

## 2. Related Work

The electrical network [8] is an important economic aspect vulnerable to severe security threats and new confidentiality issues and vulnerabilities appear related to smart network infrastructure.

### 2.1. Protocols for the Smart Grid

Many protocols have been nominated for the smart grid in the literature, especially for communication over a public network. The most prominent ones are Message Queue Telemetry Transport (MQTT) [9], Constrained Application Protocol (CoAP) [10], Common Object Request Broker Architecture (CORBA), Open Platform Communications United Architecture (OPC UA) as well as a few less-used examples such as Data Distribution Services (DDS), and Zero Message Queue (ZeroMQ). In addition, several protocols have been defined for dealing with the demand response in the smart grid. Among these we can mention OpenADR, that is an information exchange model for communicating price and reliability information to large commercial and industrial facilities [11]. IEC 61850 can also be used both for demand response and, more generally, for communication over a smart grid infrastructure.

### 2.2. Access Control in the Smart Grid

The process of data acquisition and processing to conduct further control procedures entails industrial and information technology equipment (which is integrated across the entire infrastructure) as well as the correct usage of devices and resources by all the stakeholders involved. At the same time, the increasing complexity of the smart grid architecture for the retrieval of metering [12] information and the consequent control of the electricity generation has favored the appearance of cyber-security attacks that may jeopardize the availability of resources and hence put the stability of the grid at risk.

In this complex environment, access control is essential to manage the permissions of all users, processes and heterogeneous devices that continuously interact within the infrastructure.

There are many ways of ensuring access control in ICT systems in general, including smart grid systems. One of the simplest ways is to provide role-based access control (RBAC) [13] which provides access to smart grid resources or information based on user roles, specifically defined for smart grid operation. As opposed to RBAC, attribute-based access control (ABAC) [14] draws on a set of characteristics called “attributes” which include user attributes, environmental attributes, and resource attributes, thus having a much greater number of possible control variables than RBAC.

Several access control schemes have been designed in smart grid systems over the last few years: they range from pure RBAC [15] or pure ABAC [16] to using combinations of RBAC and ABAC [17]. It is noteworthy to mention that many works mention authentication together with access control and, while authentication is subject to more advanced mathematical algorithms, usually access control is merely policy-based (such an example can be found in [18]).

### 2.3. The SealedGRID Project

The SealedGRID project copes with three significant smart grid challenges:

- Scalability: smart grid utilities can handle a variation of smart meters, but it is vulnerable to intruders, therefore the whole distribution system is in danger if someone finds a breach.
- Interoperability: smart grid protection will handle inter-domain security issues existing between nodes responsibly, with more policies and services provided.
- Trust: Soon, smart grid nodes will be available to customers, protecting them from malicious users that want to physically modify the hardware and software components to intercept personal information or to modify the cost information and energy measurements data.

SealedGRID aims to design, analyze, and implement a scalable, highly trusted and interoperable smart grid security platform with an integrated and multi-disciplinary interface which provides:

- Strong authentication.
- Attribute-based access control.
- Role-based access control.
- Anonymous attestation mechanism.
- Trusted execution environment.
- Digital certificates.
- Web of trust and blockchain.

## 3. Architecture Description

**Figure 1:**
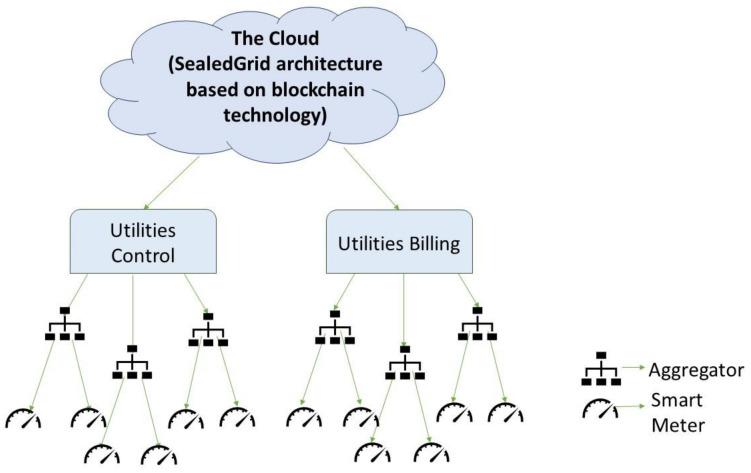
SealedGRID conceptual functional architecture.

The component entities of the proposed functional architecture behind the smart grid, partially presented in Figure 1, are the following smart grid devices: smart meter, aggregator, and utility, together with the adversary parties involved in the SealedGRID scenario, which will affect the normal execution of access control procedures: the external adversary and the insider adversary.

The smart meter [19] is responsible for collecting electricity consumption readings, and their number in each building varies, depending on its size.

Aggregator nodes are placed between utilities and the smart meters, and they are responsible for summing the individual readings received by the smart meters and then transmitting the result to the corresponding utility. In this way, the processing is distributed among the aggregators and utilities without overloading any utility. In some cases, because the architecture might not include explicit entities that perform intermediate aggregation, smart meters can play this role.

The utilities accumulate high-frequency aggregated values. They can either use these values to demand response (e.g., control the electricity consumption in a certain area) or to sum these responses and add them to the total grid consumption. It is also responsible for billing, by computing a customer’s total consumption at the end of a billing period (e.g., one month) with the use of the low-frequency meter data method.

The external adversary to the SealedGRID domain aims to destroy the domain, and to break through the customer’s privacy or endanger the system’s accountability, availability, integrity, or confidentiality.

The insider adversary is part of the SealedGRID’s domain. It can complete the authentication and authorization processes with the platform, and then it aims to violate the customer’s privacy or to endanger the system’s accountability, availability, integrity, or confidentiality.

**Figure 2:**
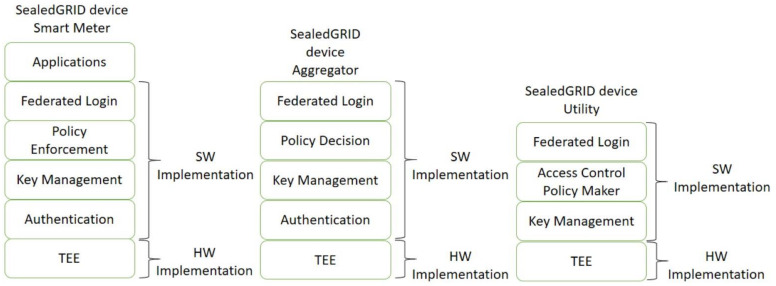
SealedGRID components and their functions.

In Figure 2, a detailed description with the required functionalities of each component is provided.

**Figure 3:**
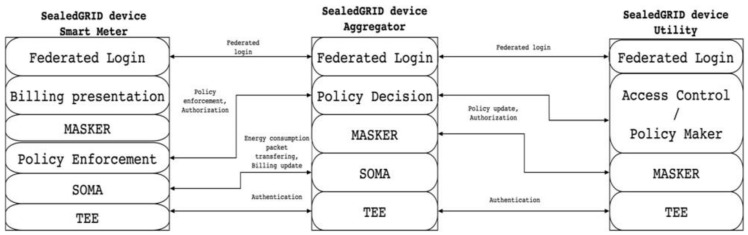
The main architectural components with the modules that comprise each component.

In Figure 3, a detailed description of each SealedGRID component, its modules, and how they interact with other components is provided. Smart meter and aggregator connection is authorized. On the smart meter side, there is policy enforcement with which the aggregator makes a decision. The smart meter and aggregator connect by federated login. Within the SealedGRID authentication component, information about energy consumption and billing updates are transferred. The trusted execution environment (TEE) handles authentication. The aggregator sums the individual readings received by the meters and transmits the result to the collector.

## 4. Authorization Component Methodology

### 4.1. Security Policy Considerations

In the smart grid scenario when different domains are interconnected to each other and collaborate, we decided to implement authorization frameworks based on the presence of policy information points (PIPs), policy enforcement points (PEPs) and policy decision points (PDPs). These entities have different responsibilities in the authorization procedure (i.e., the decision of whether granting access to a resource that has been requested):

- Policy enforcement points (PEPs) [20] are used by devices or processes to request different resources of the system, intercept and then forward an authorization request to the PDP.
- Policy decision points (PDPs) will make the decision of whether permitting or denying the access whose request has been received, and once the decision is taken, the PEP is able to permit or deny the access. Therefore, the PDP’s responsibility is to design the access control policy and to manage authorization between domains.

All SealedGRID devices are considered as Policy information points (PIPs) in which they associate a set of attribute values to the existing resources (e.g., smart meters) depending on the context information.

The extensive use of the internet as a platform for accessing distributed services creates a significant amount of personal information, a corresponding uncertainty and demand from consumers, and regulations for solutions that give users some control over their data. In SealedGRID, we designed a transition from role-based access control (RBAC) to attribute-based access control (ABAC). Thus, within SealedGRID, we considered the use of eXtensible Access Control Markup Language (XACML) for devising RBAC policies. XACML [21] is based on XML, which is a language for access control, standardized by the OASIS consortium. The language supports most security policy languages and has standard extension points for defining new functions, data types, and policy combination logic, amongst others. XACML specifies, in addition to the language, an architecture for policy assessment and a communication protocol for message interchange. Although XACML is widely used and regarded as a reference solution, it does not support privacy features, accurate evaluation, or the credential specification constraints in policies. XACML provides a fine-grained authorization method as it specifies the requirements and variables in an access control policy used to authorize access to a certain resource. Two main issues need to be resolved: first, access control operates even if interacting parties want to reveal less or no information about themselves. Second, data which have been collected or released during the access control process, as well as data which have been stored by various parties may contain sensitive information to which privacy policies (data handling) must be applied and should thus be protected. The objective is to establish a versatile framework that addresses XACML’s advantages regarding the access control and scalability.

The challenge arises when mapping the entities of the SealedGRID system to the components used to specify XACML policies, since in XACML [22], there are four categories defined:

- Subject: defines who or what demands access for an asset
- Resource: the information asset or object, determined through the action
- Action: represents the action the subject intends to do.
- Environment: defines the context related to the requested access.

For the actual formalization of the access-control rules, we based our work on the IEC 62351 standard [23], in order to follow a common framework of policies applied in the smart grid context. This is a reference in the sector to address the security of industrial networks, since it provides useful guidelines for introducing aspects related to authenticity, confidentiality, and integrity in the communication and control protocols of the smart grid. It is composed of eleven parts, where part eight is especially applied to control access mechanisms based on RBAC [8,9,10,11,12,13,14,15,16,17,18,19,20,21,22,23].

More specifically, this standard specifies a minimum set of roles to be supported in an industrial scenario with pre-defined rights: viewer, operator, engineer, installer, security administrator, security auditor, and RBAC manager (this latter can be considered as a sub-role of the security administrator), together with a list of private roles defined by an external agreement (e.g., used for certain SealedGRID purposes in specific) and another reserved list for future roles to be defined by the IEC standards.

Within this project we made this exercise and defined the XACML entities, therefore Table 1 presents a part of the entities defined, and the values for roles and actions have been identified according to roles and rights belonging to IEC-62351-8. Our authorization component utilizes the defined entities in Table 1 to implement XACML policies for the SealedGRID platform.

### 4.2. Implementation

#### 4.2.1. Authorization Component

This section describes an implementation of SealedGRID XACML-based policy enforcement points as well as policy administration points (PAPs), and in this case, both the PDP and PAP implementations rely on the open source AuthzForce Server [24], which is part of a FI-WARE system. AuthZForce provides a multi-tenant RESTful API (application programming interface) to PAP and PDP which support ABAC and is fully compliant with OASIS XACML 3.0 standard.

We made use of AuthzForce which is a FI-WARE Generic Enabler (GE) that provides open interfaces to application developers (APIs) as well as support interoperability with other GEs and we applied AuthzForce Server to comply with the SealedGRID purposes. While it is possible to use the core (community edition) [25] or just the RESTful PDP [26], we observe that it is only sufficient to provide further developments of the SealedGRID ecosystem and interoperate with other potential FI-WARE GEs.

The deployment of the AuthzForce XACML Server is detailed in this section: all access control decisions are given by AuthzForce which will read the ruleset from a previously uploaded policy domain. Note that since all interactions between the elements are initiated by HTTP (Hypertext Transfer Protocol) requests, the entities can be containerized and run from exposed ports, and the AuthzForce container is listening on port 8080, where it receives requests to make PDP decisions. A volume has been exposed to upload a pre-configured domain so that a set of XACML access control policies has already been supplied.

The following example shows the request of a decision based on a policy stating that a user with the role Operator is allowed to control the resource smart meter.

Request: to request a decision from AuthzForce, an entity (which can be a simple curl script, for now, but in the end will be a SW component) has to make a POST request to the PDP endpoint. In this case, the user has to request the access to control the smart meter, as show in Box 1:

Box 1 **Request meassage.**

curl -X POST \

http://X.Y.X.W:8080/authzforce-ce/domains/sZANABLLEeq_8QJCrBIBDA/pdp \

-H ‘Content-Type: application/xml’ \

-d ‘<?xml version=“1.0” encoding=“UTF-8”?>

<Request xmlns=“urn:oasis:names:tc:xacml:3.0:core:schema:wd-17” CombinedDecision=“false” ReturnPolicyIdList=“false”>

<Attributes Category=“urn:oasis:names:tc:xacml:3.0:attribute-category:**resource**”>

<Attribute AttributeId=“urn:oasis:names:tc:xacml:1.0:resource:**resource-id**” IncludeInResult=“false”>

<AttributeValue DataType=“http://www.w3.org/2001/XMLSchema#string”>**smartmeter**</AttributeValue>

</Attribute>

<Attribute AttributeId=“urn:sealedgrid:xacml:2.0:resource:**sub-resource-id**” IncludeInResult=“false”>

<AttributeValue DataType=“http://www.w3.org/2001/XMLSchema#string”>**operator**</AttributeValue>

</Attribute>

</Attributes>

<Attributes Category=“urn:oasis:names:tc:xacml:3.0:**attribute-category:action**”>

<Attribute AttributeId=“urn:oasis:names:tc:xacml:1.0:**action:action-id**” IncludeInResult=“false”>

<AttributeValue DataType=“http://www.w3.org/2001/XMLSchema#string”>**control**</AttributeValue>

</Attribute>

</Attributes>

…

</Request>‘

**Response**: as expected, the server returns a decision, as it can be seen in Box 2, in this case of type: “Permit”:

Box 2 **Response message.**

<?xml version=“1.0” encoding=“UTF-8” standalone=“yes”?>

<Response xmlns=“urn:oasis:names:tc:xacml:3.0:core:schema:wd-17” …>

<Result>

<Decision>**Permit**</Decision>

</Result>

</Response>

#### 4.2.2. Integration with Authentication Component

The authentication component provides a token for achieving authentication based on a distributed and scalable authentication and key management scheme called SOMA. The token provided by SOMA [27] must be validated by the authentication component, therefore, the authentication component proceeds to send a request to the AuthZ server containing the value of the Environment Category corresponding to the value of the SOMA certificate validity (whether it is true or false).

The environment attribute is called is-soma-present and, if present, must be true, for the request to be validated.

SOMA has been implemented in an isolated simulation environment; thus, it cannot be directly connected to the present implementation. Therefore, instead of just providing the authentication module with a certificate for verification, we indicatively include some steps of the creation of the certificate in order to show the process performed at the SOMA entities.

In this module we utilize the key pair of an Introducer node, and the public key of a normal node. The attributes of the normal node, along with its public key are hashed before being signed with the introducer’s private key. At this point, the essence of a SOMA certificate has been created and any node with access to the introducer’s public key will be able to verify that the hashed public key and attributes of this specific node are endorsed by an empowered entity in the network.

Depending on the verification outcome, the corresponding JSON request will be performed, so it can be seen in the requests that are presented in more detail bellow. We have not provided the part of the XACML payload indicating the resource, action and subject as they are identical to the example in Section 4.2.1.

In Box 3, a request sent to AuthZ server when the SOMA Certificate is valid can be seen:

Box 3 **Valid certificate request.**

curl -X POST http://83.212.239.224:8080/authzforce-ce/domains/sZANABLLEeq_8QJCrBIBDA/pdp -H ‘Content-Type: application/xml’ -d

‘<?xml version=“1.0” encoding=“UTF-8”?>

…

<Attributes Category=“urn:sealedgrid:xacml:2.0:attribute-category:**environment**”>

<Attribute AttributeId=“urn:sealedgrid:xacml:2.0:environment:**is-soma-present**” IncludeInResult=“false”>

<AttributeValue DataType=“http://www.w3.org/2001/XMLSchema#string”>**True**</AttributeValue>

</Attribute>

</Attributes>

</Request>‘

Box 4 shows the response received from the server:

Box 4 **Valid certificate response.**

<?xml version=“1.0” encoding=“UTF-8”?>

<ns4:Response xmlns:ns4=“urn:oasis:names:tc:xacml:3.0:core:schema:wd-17” …>

<ns4:Result>

<ns4:Decision>**Permit**</ns4:Decision>

</ns4:Result>

</ns4:Response>

The above presented show that it is possible to integrate an authentication component based on a key management scheme with a policy-based authorization component.

## 5. Trusted Computing Component

The SealedGRID authentication component described in Sec. 4 makes use of critical data and performs processes upon which the network’s security is built. More specifically, private keys and revocation certificates are securely stored inside a trusted environment that cannot be tampered either physically or digitally. Moreover, operations related to key management, like certificate signing and revocation, need to be performed in such a secure environment to ensure that the private keys used in the process are not exposed and taken advantage of by malicious parties. Remote attestation, which is a crucial part of maintaining a secure and trusted authentication component, is also executed in a trusted environment. Remote attestation is a powerful operation that, if misused, may result in denial of service, or infecting the network with malicious nodes and affect the infrastructure’s security. Therefore, we implemented a solution that provides a threefold functionality with a component for cryptographic operations, a component for secure storage and a component that will allow the integrity checking of binaries before they are allowed to run, detailed next.

Based on [7,27], we have defined the requirements for the SealedGRID trusted component. The axes, as we have selected them, are the following:

- Applicability: The applicability of the technology is basically its ability to be deployed seamlessly and organically within the SealedGRID smart meter. The core considerations here, are the fundamental capability of the trusted computing technology to be deployed in a low-powered device, typically composed of a small SoC similar to various IoT devices, with minimal architectural changes and the smallest possible costs.
- Functionality: The functionality provided by the trusted computing technology is of essence when it comes to building the functional entities of the SealedGRID trusted component. This requirement axis is concerned with the ability of the technology underlying the basic building blocks for the correct and functional orchestration of the entire trusted component.
- Performance: The performance requirements will provide insights for the considerations made when it comes to the performance overhead of the selected solution. These requirements will mostly consist of timings and computational restrictions made by the underlying hardware of the SealedGRID smart meter. Although the performance requirements are essential, they are not as binding as the applicability and functionality requirements since performance overheads that are not crippling the functionality of the device could be accepted so as to satisfy both of these crucial requirements.

We decided to utilize the TrustZone TEE [28] for the SealedGRID platform, since it provides a wide range of functionality in isolated environments. Also, TrustZone is suitable for the use in low powered devices (IoT, smart meters etc.). It is also available in the latest ARM-M architectures (Cortex-M23 and above), meaning it offers the newest benefits. We note that the current work does not aim to compare the available trusted computing implementations. Moreover, TEE offers safe execution of authorized security software, known as trusted applications. It provides end-to-end security and execution isolation by enforcing protected execution of securely verified code, confidentiality, authenticity, privacy, system integrity, and data access rights. Hence, TEE will provide a secure platform for storing and handling sensitive user information on the smart meter device. It also defines a distinction between the ‘normal world’, where common OS and applications are executed, and the ‘secure world’, which hosts trusted OSs and applications. In the proposed platform, only a subset of the smart meter functionality will be executed inside the secure world (such as storing secret keys in TEE secure storage), while the rest of the operations will remain in the normal world to maintain a minimal trusted computing base and maintain the least possible attack surface which could expose the sensitive data stored within the TEE. The communication between the normal world and the secure world will be achieved by utilizing the TEE client API, as it is defined in the GlobalPlatform specification.

### 5.1. Trusted Execution Environment Architecture in SealedGRID

TEE provides an isolated environment in which specific operations can be offloaded to and executed in a secured manner separated from the possibly compromised normal OS. Essentially, the TEE is a separate world (equipped with its virtual CPU and memory) in which these sensitive operations can reside with minimal capabilities. Given this, it is of utmost importance to maintain the minimum possible code running within this world to avoid threats and problems that follow large applications as code-based vulnerabilities and performance issues. It is measured with a term called ‘trusted code base’ (TCB), which is practically a measure of how much code is running in the secure world either in the form of secure world applications (trusted applications—TAs) or of the secure world operating system (Secure OS). The concept of TCB minimization is further defended by most TEE implementations that provide a small library set for the TAs to utilize cryptographic, arithmetic, and secure storage operations.

In this work, we aim to align with this practice by provisioning TAs that implement only the required and most sensitive components of the platform functionality. We aim to develop functionalities that can and will be reused at different points in the different components of the SealedGRID entities [29,30]. This way, we aim to provide a minimal, efficient, and as-secure-as possible trusted computing component implementation that can provide only the essential functionalities. More specifically, we aim to utilize secure storage and cryptographic operations, and we also aim to implement a secure execution environment which it will check the integrity of an entire binary before it is allowed to be executed. The above-introduced functionalities are further analyzed below:

Secure Storage, provided by the TEE, utilizes measures of cryptography and virtual separation (bit flagging by the memory management unit so to provide separate address space for the normal and the secure world). In SealedGRID, we utilize it to securely store sensitive data, certificates, and cryptographic keys that will only exist within the secure world and never leave it. Through this, we maintain the highest possible level of security for this sensitive data and prevent attackers from attempting to eavesdropping it if it were to ever leave the secure world.

Cryptographic Operations, provided by the TEE, uses internal libraries to implement a wide variety of cryptographic primitives and key generation functions to provide a seamless operation of normal world applications that utilize TAs to perform these sensitive functions. Additionally, the cryptographic functionality is interconnected with secure storage, so it enhances our scheme of having sensitive keys never leaving the secure world and being safely handled within it. Essentially, we provide an API to the normal world which will be able to specify the cryptographic operation to be executed and/or the key ID to be used with shared memory objects and attributes that will allow us to essentially provide a drop-in replacement for every cryptographic operation that would otherwise be executed in the normal world application.

Secure External Execution is a system for secure integrity checking of core applications that either use or do not use the provided previously mentioned functionalities. It builds an added layer of security for core functions of SealedGRID as the authentication and authorization component and any other native binary identified to be of higher sensitivity. The way it will work is the following: it will allow the execution of the pre-specified binaries only if they are first checked for their integrity using the TEE. Integrity check will be achieved by storing in the secure world the cryptographic hashes for each of these binaries, and before their execution, the measured hash will be compared with the stored hash, and only if they match the execution will they be allowed.

#### 5.1.1. Component Flow

**Figure 4:**
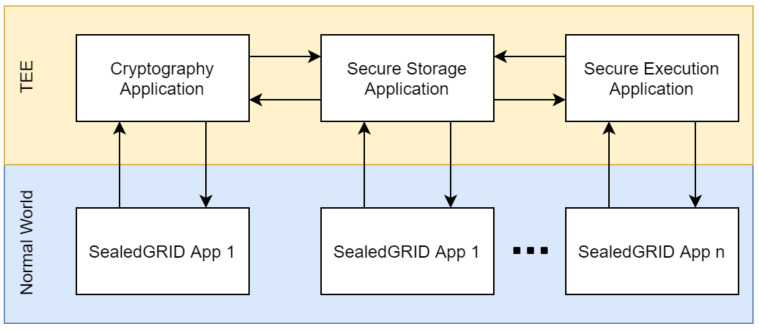
Component Overview of the TEE module.

The trusted execution environment component will be composed of a dynamic interface that will be able to serve a variety of security-sensitive functions. As discussed above, the three main functional categories target the secure storage that TEEs can provide, the secure execution of critical cryptographic functions and our implementation of binary integrity checking that will be used for the authorized execution of said binaries. All these sub-components have internal dependencies between them and thus can and will utilize functionalities of each other. For example, the cryptographic functionality will utilize the secure storage for storing the cryptographic keys used, and the binary integrity checking functionality will also use the secure storage to store a list of authorized binary hashes that can run within the normal world. This design aims to provide a dynamic secure environment that can be utilized by any application within the SealedGRID ecosystem and protect them in their core functionalities while maintaining a balance between functionality and TCB minimization. Figure 4 presents the overall scheme, where each sub-component utilizes functions of each other, and each sub-component can get used by any SealedGRID application (App).

**Figure 5:**
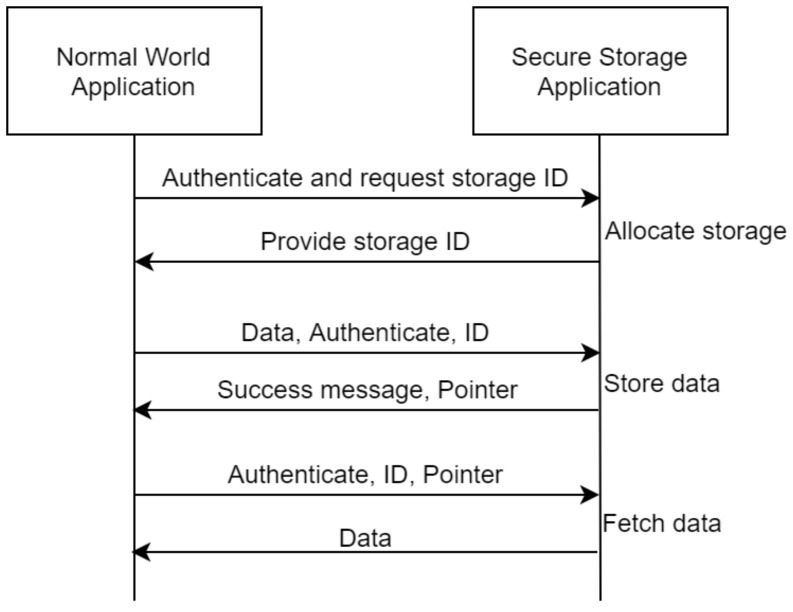
Abstract overview of the secure storage application.

The secure storage component application will provide a flexible API for the storage of security critical information which could include authentication data and smart meter data. The main concept behind this, is the utilization of storage identifiers which will allow for easy management. More specifically, a client application needs to first be authenticated and then request the allocation of a single storage slot, the secure storage application will then allocate the requested slot and return to the client application the storage ID. With the storage ID, the client application will be able to store and retrieve data within this slot given that it is always authenticated and that it provides the correct storage ID. The entire procedure is demonstrated in Figure 5 and it is to be noted here that the initial storage allocation can be enriched with additional properties which could for example indicate that a specific data slot is to be written only once, or that it cannot be retrieved to the normal world and can only be utilized in the secure world (such as cryptographic keys used for cryptographic functions executed within the TEE).

**Figure 6:**
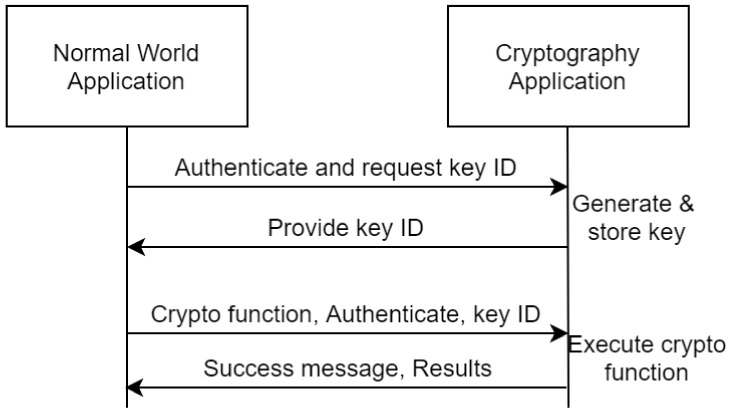
Abstract overview of the TEE Cryptography Application.

The cryptography application provides a drop-in replacement for a variety of cryptographic operations including key generation, encryption-decryption and any other operations supported by the underlying environment which is aligned with the functionality provided by the standardization TEE body GlobalPlatform in the internal API specification [29]. As apparent, this provides a two-sided functionality, one for key creation where the client application will be first authenticated and will be able to either create keys either for inner-TEE cryptography or to create external keys to be exported out of the TEE for other applications. While the second part of the cryptographic functionality is composed of the actual cryptographic functions where each function requires an optional key and the appropriate configurations from an authenticated client application. The application will be able to send: (optional) data, (optional) key ID stored in the secure storage, (optional) key stored by the application, cryptographic function ID and cryptographic function configuration. After a correct evocation of the cryptographic function, the TEE will send the success/failure message and the applicable results. Figure 6 depicts the design.

**Figure 7:**
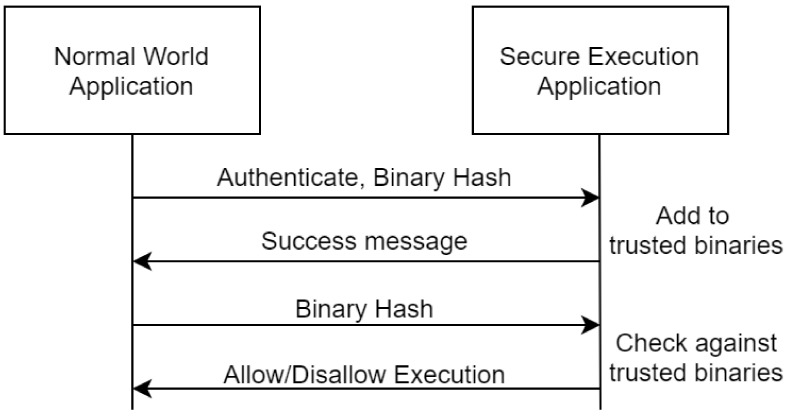
Overview of the secure execution function.

The secure execution function will be able to provide a level of assurance behind a normal world application before it is allowed to be executed. A high-level overview is depicted in Figure 7 where two main functions can be observed: (i) the registration of a trusted application through its calculated hash and (ii) the integrity checking of an already registered application that will allow or disallow the execution of it. The entire procedure will be supported through secure hash functions and possibly asymmetric cryptography for external attestation purposes.

#### 5.1.2. Integration between Modules and Related Workflows

According to the identified use cases [30] of trusted computing within the project, the basic functionalities that need support from this module are the identity validation/authentication process and the secure storage of the collected digital evidence. Given the flexibility of the proposed architecture, it can support these functionalities and any other future-identified functionality as it will provide drop-in replacement APIs for the required functionality (see Figure 4).

**Figure 8:**
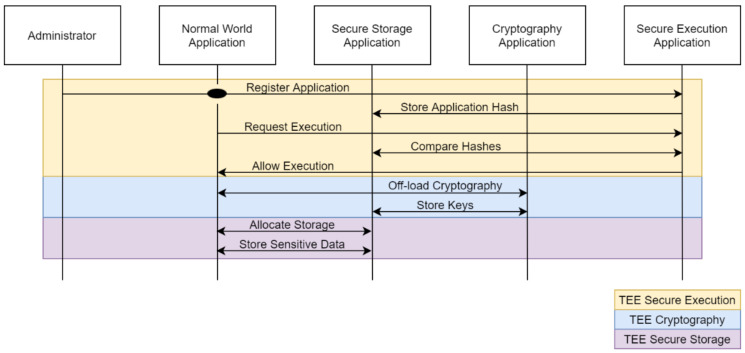
Basic use case of the full TEE functionality—secure execution, cryptography and secure storage.

We assume an administrator, who aims to execute a specific sensitive application with the utilization of the security guarantees provided by the proposed TEE architecture. First, he authenticates with the TEE and sends a cryptographic hash of the application he wants to register with the TEE secure execution function. After the TEE validates the authentication credentials provided by the administrator, it stores the cryptographic hash of the application by utilizing the TEE secure storage application, effectively registering the application in the trusted binaries registry. Once the aforementioned processes are completed, whenever this application is allowed to be executed, its hash is calculated and compared with the stored hash value within the TEE and only if the hashes match its execution can continue. With this in place, the verified application can proceed to off-load all cryptographic functions to the TEE cryptography application that can be used for key provisioning and execution of almost every current cryptographic scheme. Whenever such a functionality is needed, the application calls the provided TEE API and waits for the TEE cryptography application to respond with the results. Moreover, the application can utilize the secure storage application so that it can allocate storage within the secure world which it can use to store sensitive data in the isolated memory of the TEE. This use case is demonstrated in Figure 8.

#### 5.1.3. Design and Architectural Goals and Guidelines

The design of the overall solution is meant to be secure, minimal, and flexible, aiming to provide high functionality while maintaining a small attack surface through TCB minimization. As discussed above, the initial design is to provide a triaxial functionality within the secure world of the TEE module that strikes a balance between functionality and the deployment of the smallest possible code base within the TEE. The goal behind this design is to provision usable and secure solution to the SealedGRID project that will work as a flexible security enhancement tool which could be deployed almost as a drop-in replacement to already existing normal world functions.

The targeted functions are three: (i) cryptography, (ii) storage, and (iii) execution integrity. These three functions are common attack surfaces that often are protected through traditional security measures that we aim to enhance by utilizing the security guarantees of TEEs. Cryptography functions run within the TEE take advantage of the protected and isolated environment that will allow them to be executed securely, minimizing the possibility of inference from malware residing in the normal world. On the other hand, the storage functionality is backed by the secure storage capabilities of the TEE that allows for an isolated and cryptographically secure storage of information. Furthermore, secure storage allows for the protection of the cryptographic keys that never leave the TEE, providing a completely isolated environment for cryptographic operations. Both these benefits apply to the execution integrity checking function that can safely compare the integrity hash values of the running applications, while having a secure place for storing these hashes in the secure storage.

**Figure 9:**
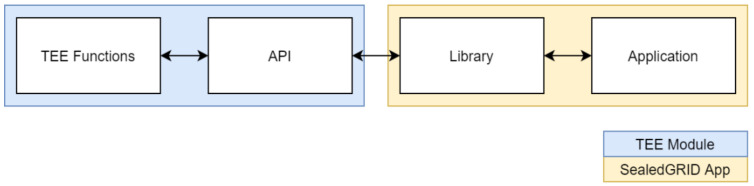
The proposed model of the segmentation between TEE and application development.

The guidelines we provide for the utilization of the TEE module follow our design and architecture; that is, security, minimality, and flexibility. We aim to provide an API on which developers can be based to program drop-in replacement libraries for the programming languages that they use. Effectively, we propose a segregated model, in which the development of the described functionalities and the API falls in the development of the TEE component and the library that utilizes the API alongside the application is developed by each application development team (see Figure 9).

### 5.2. Trusted Computing in SealedGRID Ecosystem

In the heart of the SealedGRID platform lies the TEE, because the authentication and the authorization component demand its existence for ensuring not only trust but also privacy among the entities.

The SealedGRID authentication component [31], makes use of critical data and performs processes upon which the network’s security is built. More specifically, private keys and revocation certificates are securely stored inside a trusted environment that cannot be tampered either physically or digitally. Moreover, operations related to key management, like certificate signing and revocation, need to be performed in such a secure environment to ensure that the private keys used in the process are not exposed to be taken advantage of by malicious parties. Remote attestation, which is a crucial part of maintaining a secure and trusted authentication component, is also executed in a trusted environment. Remote attestation is a powerful operation that, if misused, may result in denial of service, or infecting the network with malicious nodes, affecting the infrastructure’s security.

Moreover, trusted computing plays an essential role within the SealedGRID authorization component [32]. TEE TrustZone is the technology chosen to implement trusted computing mechanisms in the project as was justified in previous sections. The TEE can be used by SealedGRID to enable the secure handling of confidential information on devices and on server infrastructure. Among the most relevant features, we have mentioned the isolation. The TEE standard creates an isolated environment that runs in parallel with the operating system, providing security for the rich environment. This isolation is essential to implement the SealedGRID authorization component that keeps sensitive information that will be securely stored within the secure zone in TEE as the authorization token. Isolation can be used to store and encrypt (if applicable) sensitive information from context awareness processed to implement SealedGRID authorization components.

### 5.3. Trusted Computing Component Implementation

Following the defined architecture, we implemented a solution that provides a threefold functionality with a component for cryptographic operations, a component for secure storage and a component that will allow the integrity checking of binaries before they are allowed to run. The cryptography and secure storage components are stand-alone and can be used and configured dynamically from other applications by calling the corresponding executable binary with the appropriate flags and configurations. While the integrity checking component depends on the other two to enroll binaries (hash, sign and securely store the signature of the binary to be enrolled) and check binaries (pull signatures from secure storage, hash the binary and verify it) with its functionality being mainly targeted towards system administration tasks.

#### 5.3.1. OP-TEE

For the actual implementation of the SealedGRID trusted computing component, we chose the OP-TEE project [33] that provides a fully functional secure world operating system and the corresponding normal world hooks that allow for normal–secure world communications. Also, it provides a toolset for the development of trusted applications which is GlobalPlatform compliant and allows for portable code for other compliant systems. The project consists of the optee_os which is the operating system of the secure world and contains libraries that implement GlobalPlatform-defined TEE functionalities with minimal extra libraries that mainly target providing support for common operations that take place within a TEE. On the other hand, the optee_client contains libraries and APIs for the correct function of the normal world side of the TEE, something that includes proper system calls, GlobalPlatform compliant API support of the normal world client applications and proper authorization to the TEE device driver. The build component of the OP-TEE project contains all the necessary build recipes for setting up the entire environment for a variety of targets (development boards and emulated QEMU targets) as well as for building complete trusted and client applications. Underneath the aforementioned operating systems, the OP-TEE project utilizes a standard ARM TrustZone firmware [34] that acts as a *hypervisor* between the normal and the secure world. This firmware is essentially tasked with handling any TEE-related system calls or interrupts and managing the TEE so as to provide the defined functionality securely and correctly.

#### 5.3.2. Cryptographic Component

The cryptographic component is mainly concerned with implementing specific cryptographic functions in the secure environment of a TEE. The entire component is designed to be used with configuration flags that specify what exact operation should be executed, a process that begins from the normal world client applications that interprets these flags and properly calls the trusted application so that it will execute the specified function within the TEE which then in turn will return the results back to the normal world.

The cryptographic component contains some basic operations, namely:

I. key generation
II. encryption/decryption
III. hashing
IV. signing/verification.

The design of each of these aims to provide a wide range of predefined commonly used cryptographic algorithms but also being easily extensible so that new algorithms could be added. The initial architecture of this module is to be used as an external binary (the programmer will have to call it as a system program), but external libraries can be implemented to provide easier to use APIs for the specific language that the developer is using. In the following subsections we will present the functionalities implemented by each module.

The key generation module is tasked with generating keys that reside only within the TEE and never leave it and this is achieved by utilizing the internal secure storage of the TEE that holds the generated keys. These are later pulled for cryptographic operations within the secure world, and when generating a key, a user must specify first the *keygen* directive after the binary call alongside with the following parameters: (i) *Key ID*: the ID which will be used for later accessing the key. Essentially it is used as a unique identifier for the storage ‘drawer’ that holds the key; (ii) *Key Type*: the type of the key that will be generated. Valid values for this flag are RSA for asymmetric cryptographic and signing keys or AES for symmetric cryptography keys; (iii) *Key Size*: the size of the key that will be generated. This needs to comply with standard practices and will affect the type of available cryptographic functionalities that will be available with said key (RSA 1024/2048, AES 128/256). Under the hood, the client application sends all the aforementioned information to the secure world application so that it can initialize the key generation process. This is broken to the actual key generation with all the desired properties, executed with the TEE_GenerateKey function and temporarily stored on the secure world in a TEE_ObjectHandle with the TEE_AllocateTransientObject function. With the object handle in hand, the trusted application continues to permanently store the key in its secure storage. This is achieved with the utilization of the TEE_CreatePersistentObject function that takes in the desired ID of the stored key that the user has defined.

The encryption and decryption functionalities are included within the cryptographic module which is tasked with the encrypting and decrypting in the various available modes of AES for symmetric cryptography or of RSA for asymmetric cryptography. There are specific modes hardcoded in the application which can easily be modified or extended in the source code depending on the needs and requirements of the end application. These cryptographic functionalities are invoked using the compiled binary with specific directives and flag configurations with the target of being called by SealedGRID functionalities to provide secure cryptography throughout the smart meter deployed applications. The normal world application sets up all the required parameters and makes the corresponding call to the trusted application so as to clearly state the desired functionality from the TEE. In the secure world, the trusted application initially sets the encryption decryption functionality with the desired algorithm and mode, then it calls the get_key() function of the key generation module to pull the specified key and finally invokes the cryptographic operation. When the results are returned to the normal world application, the trusted application writes the output to the specified file which is created if it does not exist.

The hash function is a part of the cryptographic module of the SealedGRID trusted computing module, and it allows for hash operations of inputs with the output being returned either in a file or to the standard output of the terminal. The client specifies the desired hash operation algorithm and the input and output parameters, that the trusted application receives and acts accordingly. Available algorithms are: (i) TEE_ALG_SHA256: the SHA256 algorithm, and (ii) TEE_ALG_SHA512: the SHA512 algorithm. Under the hood, the client application sends all the relevant information to the trusted application which will in turn act upon them and execute the defined operation. More specifically, the operation mode is set to the TEE_MODE_DIGEST alongside the specified algorithm, and afterwards, the hash function is executed with the results being returned to the client application.

The signature and verification functions are part of the cryptographic component and more specifically, the asymmetric cryptography functionality. The purpose of this functionality is to provide a secure method for signing and verifying hashes that are fed into the binary either through the command line or from input files. The output signature is stored in the specified output-file and, regarding the verification, the POSIX success code of zero (0) is returned on correct verification and error codes are returned for incorrect verification. The process is similar to the asymmetric part of the encrypt and decrypt functionality and similar arguments are required for the execution for the signing and verification of hash digests. Under the hood, the client application compiles all the required information and invokes the trusted application so that it can execute the specified function. In the secure world, the trusted application sets all the appropriate modes and configuration for the asymmetric signature or verification in the cmd_do_crypto() function, pulls the defined key with the get_key() function and executes the defined asymmetric function in the RSA_Operation() function.

#### 5.3.3. Secure Storage Component

The secure storage component is a separate binary part of the SealedGRID trusted computing component that is tasked with securely storing files and data within the secure storage of the TEE. The secure storage is an encryption backed and hardware protected storage location that allows for normal world inaccessible and encrypted data. This storage location is used for holding sensitive data from the normal world as well as data that never leave the TEE as we have already established in the key generation component that creates and stores cryptographic keys that never leave the premises of the TEE. In the following two chapters we will analyze the functionality of storing and pulling data from the secure storage in the context of the normal world usage (data will travel from the normal world to the secure world and vice versa).

##### Store Data in the Secure Storage

The data storage process can be called from the secure storage binary with the store directive, which essentially allows for storing a file in the secure storage with a given ID locator that will later be used for pulling this file. The following configurations must be specified for a successful data storage alongside with the *store* directive: (i) File name: the file name of the file to be stored and (ii) File identifier: The ID of the secure storage location where the file will be stored. Under the hood, the size of the file is computed to properly set all the required parameters which are then sent to the TEE. The trusted application on the other side, creates the corresponding persistent object that is populated with the TEE_WriteObjectData() function that copies the contents of the file in the persistent object.

##### Pull Data

Pulling data from the secure storage is similar to the process of storing the data, so the client application needs to specify the identifier of the stored object and the name of the file to be created that will contain the pulled data. Under the hood, the client sends the identifier of the data storage to be pulled to the trusted application. The trusted application then pulls the data that the identifier points to with the command TEE_ReadObjectData().

#### 5.3.4. Binary Verification Component

The binary verification component is a standalone function of the SealedGRID trusted computing component that allows for the secure integrity verification of executable binaries before they are allowed to run. This component first allows an administrator to enroll binary signatures in the secure storage, a process that first calls the cryptographic component to securely digest and sign the hash to create a valid certificate and then stores the resulting certificate in the secure storage through the secure storage component. then, when a binary is to be executed, the certificate is pulled from the secure storage, the hash is recalculated through the cryptographic component which will be used once again to verify the hash in comparison with the pulled certificate. In the following subchapters, we will analyze the binary enrolment and verification.

##### Binary Enrolment

The binary enrolment process initially copies the binary in administrator-only directory to avoid any race condition vulnerabilities. It then proceeds to call the cryptographic component to produce the hash of the binary and then its certificate with the usage of the defined asymmetric key. With the certificate in hand, the final task is to store the certificate within the TEE through the secure storage component.

##### Binary Verification

The binary verification procedure is the reverse of the enrolment procedure, and it essentially allows for the integrity verification of executables before they are allowed to run. Once again, the binary is initially copied to an administrator created temporary directory so as to avoid race conditions and then it is hashed with the cryptographic component. Afterwards the corresponding certificate is pulled from the secure storage given the name of the binary. Finally, with both the certificate and the hash in hand, the cryptographic component is called once again to verify the binary given the specified asymmetric key ID. If the binary is correctly verified, it is automatically executed from the temporary directory, if not an error is returned.

## 6. Results

### 6.1. Authorization Component Evaluation

The authorization component was subjected to a stress test, using a series of scripts written in bash, as well as two tools: percentile (https://github.com/yuya-takeyama/percentile (accessed on 14 April 2021)) and ntimes (https://github.com/yuya-takeyama/ntimes (accessed on 14 April 2021)).

A series of tests have been defined, mainly by varying the number of clients (running several processes that call the REST API interface of the AuthZ component). The most relevant result that we considered is the measured API response time as well as CPU and RAM variation according to the number of clients.

#### 6.1.1. API Response Time

The number of clients that simultaneously call the API interface of the AuthZ component has been varied and then the average, minimum, maximum response time to perform a POST operation and the standard deviation but also the percentages of its different values were measured.

Percentile *x*—represents the value at which *x* percentages of the data are below the measured value.

For the purpose of this evaluation, we will consider an acceptable response time of the order of 500 ms (https://www.nngroup.com/articles/response-times-3-important-limits/ (accessed on 15 April 2021)).

**Figure 10:**
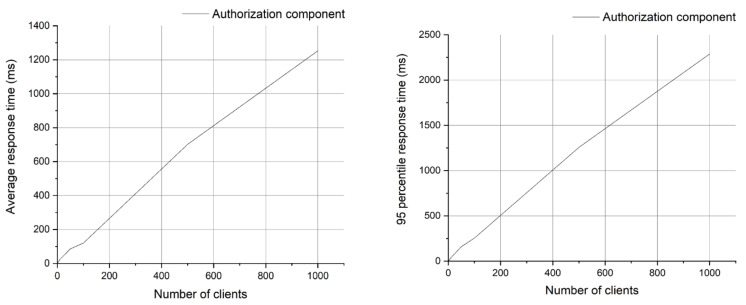
Average response time and 95th percentile response time.

Following the analysis of Figure 10, we can observe that the response time exceeds 500 ms, when the number of clients exceeds 100. Moreover, the growth rate of this response time increases with the number of customers exceeding 100 in a linear fashion. A similar trend can be observed for the 95th percentile, but here we mention that the values are much worse than the average. The conclusion is that the API interface of the AuthZ component cannot support simultaneous connections from more than 100 smart grid clients, without significant degradation of the response time. This provides valuable insight when designing the access control solution and the general architecture for a secure smart grid system.

#### 6.1.2. RAM and CPU Consumption

While running the stress tests mentioned, we also used the Linux commands *sar* (part of sysstsat (https://man7.org/linux/man-pages/man5/sysstat.5.html (accessed on 15 April 2021)) package) for CPU consumption and the values of RAM consumption reported by */proc/meminfo* file. The variation was recorded for 180 s.

**Figure 11:**
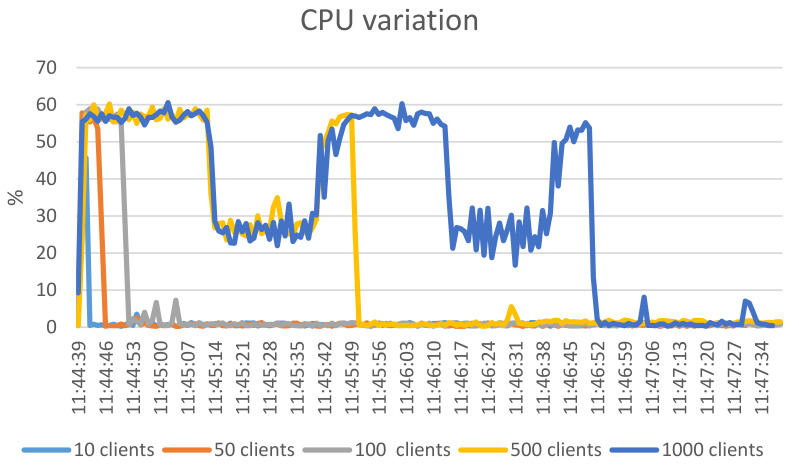
CPU consumption variation for different number of clients.

Analyzing Figure 11, we can see that the CPU consumption jumps to about 60% and remains until the concurrent API calls return. The more numerous the clients, the more the CPU consumption remains at 60%. A periodic drop to about 30% CPU for 500 and 1000 clients is noticeable. This behavior can be attributed to the thread scheduling in the Linux kernel used—some of the API calls are on hold and after some of them finish (about 60 s, which keeps CPU load at 60%), the rest that run after keep the CPU load at 30%.

Figure 11 shows that the memory consumption has a linear increase, but with little relative increase. For 1000 clients, the memory consumption jumps by 42% and then linearly rises to about 52% of the initially consumed RAM (therefore an 8.5% increase).

**Figure 12:**
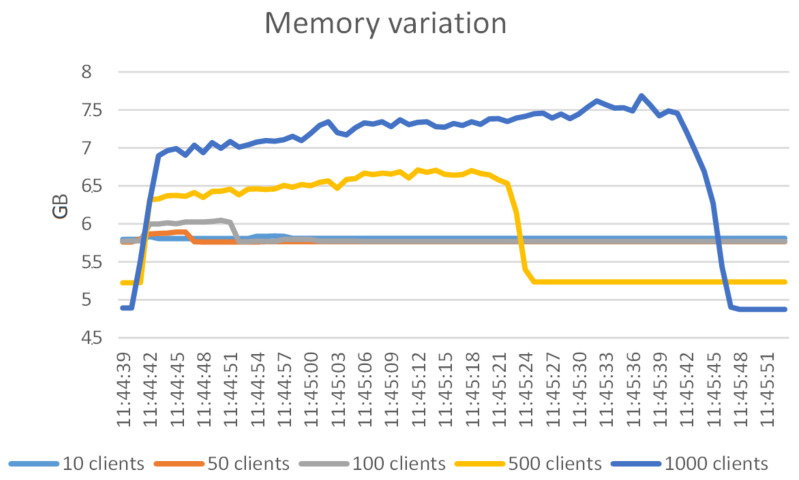
RAM consumption variation for different number of clients.

**Figure 13:**
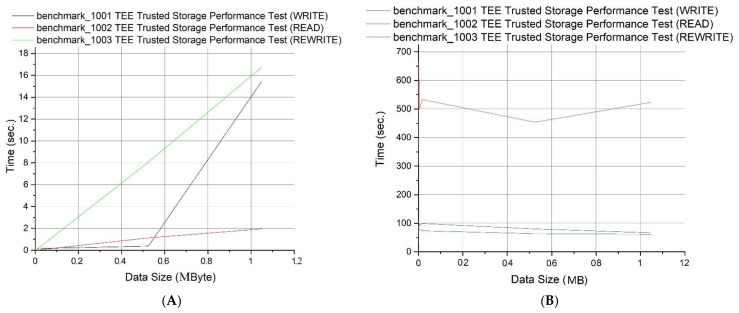
(**A**) WRITE, READ, REWRITE Tests—Data Size vs. Time and (**B**) Data Size vs. Speed.

Figure 12 shows the variation of the memory consumption only until 11:45:51. After that the memory consumption stays nearly constant and we opted not to graphically represent this part. From Figure 12 and Figure 13 we can conclude that the RAM and CPU impact can be accommodated by medium-end hardware. Corroborating with the results in Section 4.2.1, where we concluded that 100 simultaneous connections to the authorization component can be supported, we see that the impact of the authorization component when receiving multiple simultaneous API calls is negligible especially on the RAM consumption.

### 6.2. Performance Evaluation of the SealedGRID Trusted Computing Component

A series of tests were conducted using the OP-TEE test suite, so the OPTEE environment was set up on a virtual machine within a computer where resources were dedicated to it. The computer and VM have the specifications listed in Table 2, and the QEMU tool was used within the VM to virtualize an ARM processor that provides the underlying TrustZone technology that OP-TEE uses to bootstrap its secure and normal world.

We note that the viability and performance of the proposed trusted computing component reevaluated through emulation. We ran a series of benchmarks for the performance of the underlying OP-TEE primitives that our solution utilizes, the aim here is to create a concise report of how efficiently the OP-TEE framework can handle different kinds of payloads in both trusted storage and cryptographic operations, as well as to run all the OP-TEE defined regression tests to check its proper functionality.

We performed the following tests with respect to the data size: (i) data write time, (ii) data read time, and (iii) data rewrite time. We observed that the data read time increases linearly with the doubling of the data size; the read times are very small for small data sizes and finally, that the time to rewrite remains very small for small data sizes, however it increases linearly for larger ones. Figure 13A summarizes these results regarding the required time, while Figure 13B presents the speed for the aforementioned measurements regarding the corresponding data size.

Moreover, we have measured the performance of SHA1, SHA256, AES ECB and AES CBC with random inputs of the same size, and Table 3 summarizes the results of this evaluation.

Given the above measurements (see Table 2) that we produced, we can observe that our solution is capable of supporting the SealedGRID solution in its functionalities. In the context of data storage, given the fact that the stored data will be of small size (mainly cryptographic keys and signed hashes) the measured speed is more than enough to instantly push and pull data from the TEE or stage. On the other hand, the cryptographic primitives of the OPTEE platform provide performance in the order of microseconds (us) which can provide exceptional performance given the fact that it is provided by an external security module. This performance can vary depending on the platform that the software runs on (hardware accelerators) and how optimized the cryptographic libraries are. All in all, we observe the overall performance and correctness of the SealedGRID trusted computing component as more than acceptable for the targeted functionality and it meets the requirements.

## 7. Conclusions and Future Work

In this work we have presented the entire process that we followed while designing a functional trusted computing component that fulfils the requirements of the SealedGRID project, implementing it and measuring its performance. We chose the TrustZone trusted execution environment technology which is abundantly available in devices with ARM processors which are common in small devices that cover the needs of the smart meters of SealedGRID. With this in place, we selected the OP-TEE platform, a complete TEE environment that provides secure- and normal worlds with the usage of the TrustZone technology and can be used to develop both physical devices based or emulation-based TEE applications. After the selection process, we created a trusted computing component architecture that aims at providing versatility in its functionality by giving commonly used primitive functions to the user (cryptography, signatures, hashing, and storage).

The paper presented steps taken to implement XACML policies tailored for smart grids, therefore, proper XACML entities were defined according to the scope and purpose of the SealedGRID project. Also, a reference implementation of the trusted computing component was presented, and a preliminary evaluation of the implementation was performed.

## Figures and Tables

**Table 1:** Defined Entities.

Short Name	Namespace	Category	Data Type	Value Range
role	eu.sealedgrid.user	Subject	String	Operator, Auditor, Provider, Customer, Administrator, Installer, Engineer
actionId	eu.sealedgrid.action	Action	String	View, Read, Dataset, Reporting, FileRead, FileWrite, File Management, Control, Config, SettingGroup, Security
objectType	Eu.sealedgrid.object	Resource	String	SmartMeter, Aggregator, Utility
criticality	Eu.sealedgrid.context	Environment	String	Low, Medium, High
anomalyLevel	eu.sealedgrid.context	Environment	Double	0...0.01...1
communicationProtocol	eu.sealedgrid.context	Environment	String	Modbus, OPC UA, Ethernet/IP
domainId	eu.sealedgrid.domain	Subject	String	A, B, C, D

The above presented show that it is possible to integrate an authentication component based on a key management scheme with a policy-based authorization component.

**Table 2:** Testbed parameter.

Specification	Value
CPU	*Intel i7-8568u*
Number of CPU Cores/Threads	4/8
RAM	16 GB
Host Operating System	Windows 10.0.19041
VM Number of Processor Cores/Threads	2/2
*VM allocated RAM*	*2 GB*
*VM Operating System*	*Ubuntu 20.04*
*QEMU Version*	*5.0.0*

**Table 3:** SHA1, SHA256, AES ECB, AES CBC Execution Times.

Algorithm	Min (us)	Max (us)	Mean (us)	Stddev (us)
SHA1	586.14	5042.05	729.56	105.52
SHA256	731.90	2958.29	919.312	75.06
AES ECB	596.83	2811.23	749.524	89.58
AES CBC	613.408	385.5	729.82	98.48
